# *α*-Tocopherol Prevents Sperm Apoptosis and Necrosis in Rats Exposed to 2,3,7,8-Tetrachlorodibenzo-p-dioxin

**DOI:** 10.1155/2022/3685686

**Published:** 2022-02-21

**Authors:** Dewa Ketut Meles, Kadek Rachmawati, Iwan Sahrial Hamid, Imam Mustofa, Wurlina Wurlina, Niluh Suwasanti, Desak Ketut Sekar Cempaka Putri, Suzanita Utama

**Affiliations:** ^1^Laboratory of Veterinary Pharmacology, Faculty of Veterinary Medicine, Airlangga University, Kampus C Mulyorejo, Surabaya 60115, Indonesia; ^2^Division of Veterinary Reproduction, Faculty of Veterinary Medicine, Airlangga University, Kampus C Mulyorejo, Surabaya 60115, Indonesia; ^3^Department of Clinical Pathology, Widya Mandala Surabaya Catholic University, Jl. Kalisari Selatan No. 1, Kalisari, Kec. Mulyorejo, Surabaya 60112, Indonesia; ^4^Division of Cardiology, Faculty of Medicine, Airlangga University, Jl. Mayjen Prof. Dr. Moestopo No. 47, Surabay 60132, Indonesia

## Abstract

2,3,7,8-tetrachlorodibenzo-p-dioxin (TCDD) is a persistent organic pollutant that induces overproduction of reactive oxygen species (ROS). Studies on avoiding the adverse effects of dioxin pollution exposure are needed in all aspects, including reproductive health. This study aimed to determine the effect of *α*-tocopherol on superoxide dismutase (SOD) and malondialdehyde (MDA) levels, live spermatozoa, apoptosis, and necrosis in male rats exposed to dioxin as a model. Thirty healthy 12-week-old male rats were randomly divided into five groups. Rats in the control group were given corn oil twice daily at 4-hour intervals. The remaining rats were given TCDD 700 mg/kg BW daily, followed by administration of corn oil and *α*-tocopherol at doses of 77, 140, and 259 mg/kg BW/d for T0, T1, T2, and T3 groups, respectively. The treatments were conducted for 45 days; all rats were euthanized to collect blood and testicular samples on day 46. The results showed that exposure of TCDD resulted in a decrease in SOD activity and live spermatozoa and increased MDA level and death, apoptosis, and necrosis of spermatozoa (T0) compared to the control (C) group (*p* < 0.05). The administration of *α*-tocopherol, starting from the doses of 77 (T1), 149 (T2), and 259 mg (T3) per kg BW, was sequentially followed by returning MDA levels, recovering SOD activities, and restoration in the percentage of living, dead, apoptotic, and necrotic spermatozoa, similar (*p* > 0.05) to those of the control group. It could be concluded that the administration of *α*-tocopherol resolves the harmful effects of TCDD on the viability of spermatozoa in rats as a model.

## 1. Introduction

The formation of 2,3,7,8-tetrachlorodibenzo-p-dioxin (TCDD) was as a by-product in the burning processes of organic materials or a side product in organic synthesis. It was a persistent organic pollutant and a colorless solid with no distinguishable odor at room temperature [1]. TCDD induced premature senescence in neuronal cells by promoting intracellular ROS production that accelerates the onset of neuronal senescence to induce neurotoxic effects [2]. The neuroendocrine activity along the hypothalamic-pituitary-testicular axis regulated spermatogenesis [3]. A reverse set of changes in the AhR protein and mRNA response to TCDD suggested that AhR functions as a physiological regulator [4]. Our previous study indicated that TCDD depressed spermatogenic staging, number of Leydig cells, spermatozoa, seminiferous tubule diameter, and thickness of tubule epithelium [5]. TCDD caused mitochondrial dysfunction and the accumulation of cellular reactive oxygen species (ROS) [2]. Polyunsaturated fatty acids in the cell membrane, including the membrane of mitochondria, are sensitive to ROS exposure, causing lipid peroxidation. The end product of lipid peroxidation was MDA; therefore, MDA was a biomarker for oxidative stress [6].

In living beings, there are several endogenous antioxidants, including SOD. SOD is essential for protecting cells against oxidative stress [7]. The antioxidant matter can scavenge free radicals due to TCDD [8]. Meanwhile, *α*-tocopherol is an exogenous antioxidant, a fat-soluble nonenzymatic antioxidant. The antioxidant *α*-tocopherol can break the TCDD chain bond with AhR (aryl hydrocarbon receptor) found in the cell cytoplasm, thus protecting cells from lipid peroxidation [7]. *α*-tocopherol was a fertility triggering agent that can normalize seminiferous tubular epithelium [9] and had a protective effect on oxidative stress and sperm apoptosis in diabetic mice [10]. The administration of *α*-tocopherol proved to repair the damage of testicle histology in rats exposed to dioxin [5]. The decreasing spermatogenic staging due to TCDD exposure may be caused by the death of sperm due to apoptosis and necrosis. There is no report about the use of *α*-tocopherol to avoid apoptosis and necrosis of spermatozoa on rats exposed to TCDD.

Therefore, this study aimed to determine the effect of *α*-tocopherol for maintaining sperm viability, decreasing sperm apoptosis and necrosis through maintaining SOD, and decreasing MDA levels on male rats exposed to dioxin.

## 2. Materials and Methods

### 2.1. Ethical Approval

The experimental protocol of this study was approved by the Animal Care and Use Committee, Airlangga University, Surabaya, Indonesia (no. 267/HRECC.FORM/VI/2020). Experiments have been conducted with minimum pain or discomfort, following the guidelines established by the Institutional Animal Ethics Committee.

### 2.2. TCDD and *α*-Tocopherol Dosages

The dose determination of TCDD and *α*-tocopherol (both from Sigma-Aldrich, Singapore) was based on our earlier study [5]. This study used a single dose of 700 ng/kg BW/day TCDD. Meanwhile, the doses of *α*-tocopherol were 77, 140, and 259 mg/kg/day. Corn oil (Mazola®, Codaa Switzerland AG) was used as a solvent for TCDD and *α*-tocopherol.

### 2.3. Treatment of Experimental Animals

Thirty healthy male rats (*Rattus norvegigus*, Wistar strain) aged 12 weeks, weighing about 200 grams, were randomly divided into five groups equally for control (C), T1, T2, T3, and T4 group. The control group rats were administered corn oil twice a day in four-hour intervals. The remaining rats in the treatment group were given TCDD 700 mg/kg BW/day, and four hours later, they were given corn oil, *α*-tocopherol 77, 140, and 259 mg/kg BW/day for the T0, T1, T2, and T3 groups, respectively. TCDD and *α*-tocopherol were administered orally in 1 mL volume daily for 45 days, and on day 46, all rats were euthanized for blood and testicular samples. Euthanasia was performed via decapitation by trained personnel.

### 2.4. SOD Activity Measurements

The tunica albuginea of the testes was removed; then, the testes were homogenized at a ratio of one gram tissue per ml of 0.05 M Tris-HCl buffer pH 7.5. The homogenate was centrifuged at 12000 ×  g for 15 minutes to remove most of the tubules and other large sediment tissue fragments. The supernatant was centrifuged for 20 minutes at 50,000 ×  g and 150,000 ×  g for 60 minutes. The lipid layer on the surface of the supernatant was removed after each centrifugation, and the final supernatant was used for SOD activity measurement using a colorimetric superoxide dismutase activity assay kit (CS0009, Sigma-Aldrich, Singapore) at a wavelength of 550 nm [11].

### 2.5. MDA Level Measurements

Blood samples were collected from below the aortic arch for measuring serum MDA levels using the thiobarbituric acid-reactive substance (Sigma-Aldrich, Singapore) with a UV-1601 spectrophotometer at a maximum wavelength of 535 nm [12, 13].

### 2.6. Live and Dead Spermatozoa Count

Semen was collected from the cauda epididymis and diluted 200 times with 0.9% NaCl. A drop of the semen sample and a drop of eosin-nigrosin were mixed homogeneously, smeared, and quickly dried over the flame. The viability of the sperm was examined under a light microscope Nikon E200 (Nikon Corporation, Tokyo, Japan) at 400× magnification. Live spermatozoa were white and transparent on the head since they did not absorb color, while the dead spermatozoa absorbed a red-purple color [14]. The percentages of live and dead spermatozoa were determined out of the total of 300 spermatozoa.

### 2.7. Apoptosis and Necrosis Spermatozoa Count

Dry smeared semen samples were fixed with absolute methanol and glacial acid for 15 min, stained with acridine orange, and then observed with a fluorescence microscope at 100× (Nikon Eclipse E800, Tokyo, Japan). Apoptotic spermatozoa were yellow to reddish, and the necrotic ones were brownish-orange. Meanwhile, live spermatozoa were green-stained [15, 16]. The percentages of apoptosis and necrosis spermatozoa were determined out of the total of 300 spermatozoa.

### 2.8. Statistical Analysis

The data were analyzed using a one-way ANOVA followed by Tukey's honestly significant difference test at a 95% confidence level (SPSS version 23, IBM, New York, United States).

## 3. Results

TCDD exposure was followed by a decrease in SOD activity by half and an increase in MDA levels twice (T0) compared to the control group (C) (*p* < 0.05). Administration of *α*-tocopherol starting at a dose of 140 mg per kg BW restored the same SOD activity as in the normal mice (group C) (Table 1).

Different superscripts in the same column show significant differences (*p* < 0.05). Rats in the control group (C) were administered 0.5 mL of corn oil twice daily at 4 h intervals for 45 d. Rats in groups T0, T1, T2, and T3 were administered TCDD 700 ng/kg BW daily and 4 h later were administered corn oil, 77, 140, and 259 mg/kg BW daily *α*-tocopherol, respectively, for 45 d.

### 3.1. Live, Dead, Apoptotic, and Necrotic Spermatozoa

**Figure 1:**
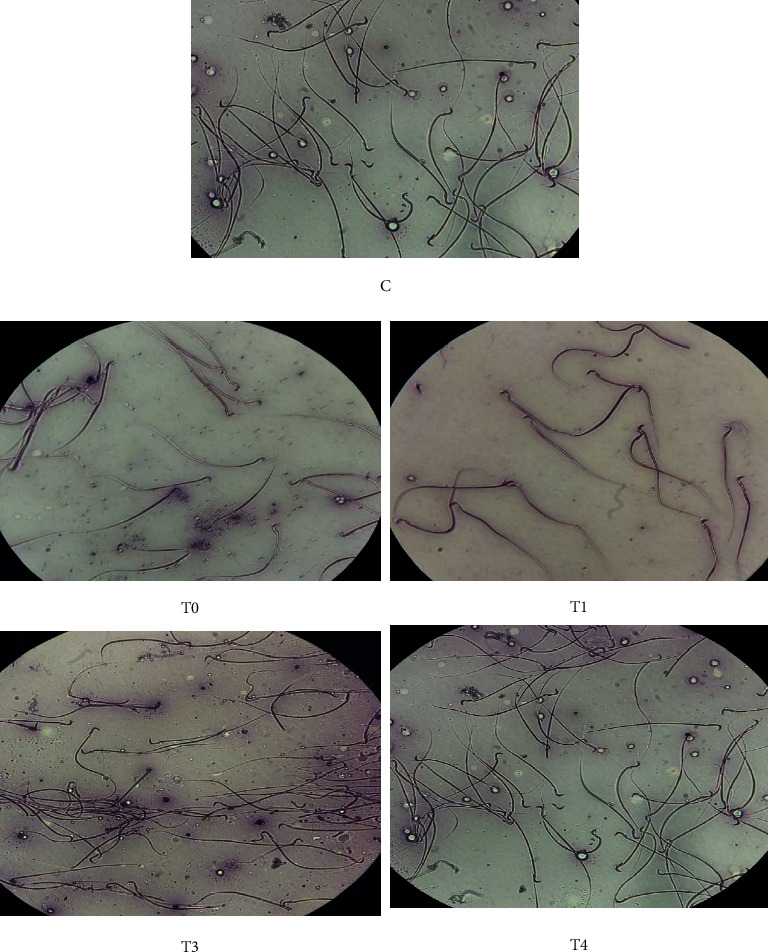
Live and dead sperm of rat exposed to TCDD and treated with *α*-tocopherol. Rats in the control group (C) were administered 0.5 mL of corn oil twice daily at 4 h intervals for 45 d Rats in groups T0, T1, T2, and T3 were administered TCDD 700 ng/kg BW daily and 4 h later were administered corn oil, 77, 140, and 259 mg/kg BW daily *α*-tocopherol, respectively, for 45 d Live spermatozoa do not absorb color on the head, whereas dead spermatozoa absorb a red-purple color from the stain (eosin-nigrosin staining), at 400× magnification under a light microscope Nikon E200 (Tokyo, Japan).

**Figure 2:**
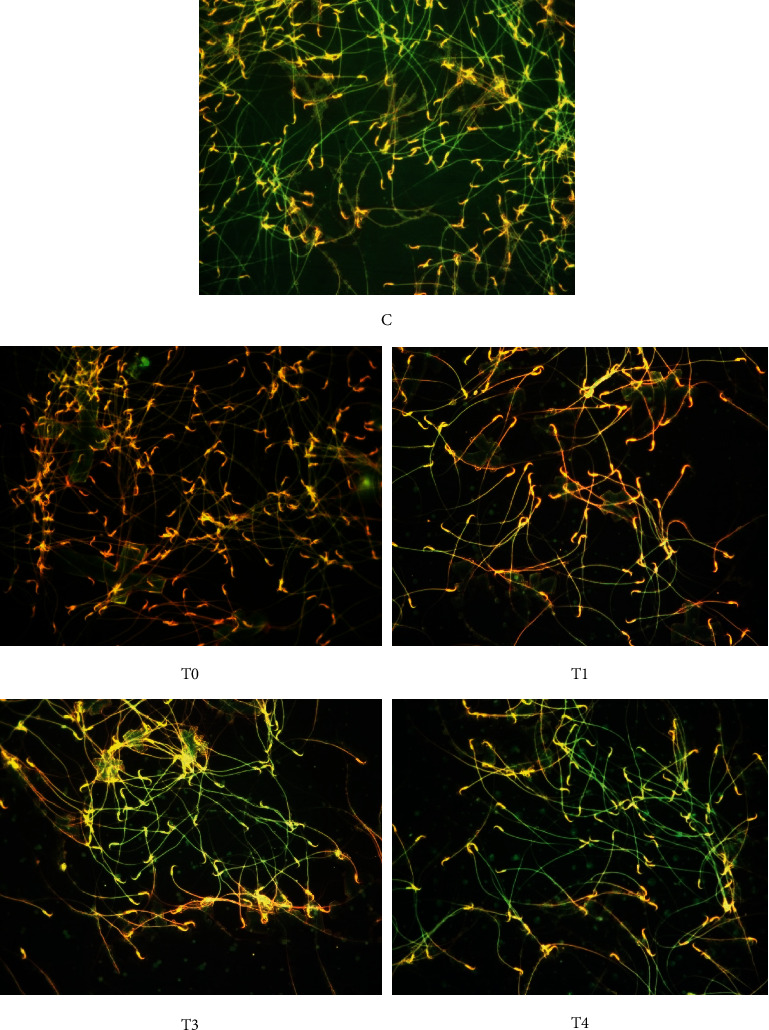
Apoptotic and necrotic spermatozoa of rats exposed to TCDD and treated with *α*-tocopherol. Rats in the control group (C) were administered 0.5 mL of corn oil twice daily at 4 h intervals for 45 d. Rats in groups T0, T1, T2, and T3 were administered TCDD 700 ng/kg BW daily and 4 h later were administered corn oil, 77, 140, and 259 mg/kg BW daily *α*-tocopherol, respectively, for 45 d. The membrane of apoptotic spermatozoa will be green with yellowish cytoplasm and nucleus, and necrotic spermatozoa will be brown. Meanwhile, live spermatozoa were green (acridine orange staining), at 100× magnification under a fluorescence microscope (Nikon Eclipse E800, Tokyo, Japan).

Rats exposed to TCDD (T0 group) showed decreases of live spermatozoa (leaving only about 40%) and 4-5-fold increases, respectively, in dead, apoptotic, and necrotic spermatozoa compared to normal rats (C group) (*p* < 0.05). Administration of *α*-tocopherol resulted in increased live spermatozoa. The dose of 259 mg *α*-tocopherol per kg BW recovered all of those parameters to those of the control group (*p* > 0.05) (Table 2 and Figures 1 and 2).

Different superscripts in the same column show significant differences (*p* < 0.05). Rats in the control group (C) were administered 0.5 mL of corn oil twice daily at 4 h intervals for 45 d. Rats in groups T0, T1, T2, and T3 were administered TCDD 700 ng/kg BW daily and 4 h later were administered corn oil, 77, 140, and 259 mg/kg BW daily *α*-tocopherol, respectively, for 45 d.

## 4. Discussion

TCDD is a toxic substance that induces oxidative stress, which is an imbalance between free radicals and antioxidants [8]. Free radicals react with polyunsaturated fatty acids, resulting in lipid peroxidation [17]. MDA was a marker of lipid peroxidation [18]. Lipid peroxidation is a degenerative process that plays a crucial role in the pathogenesis of many diseases by decomposing hydroperoxide products into cytotoxic derivatives [19]. Actually, there are many antioxidants in the living cell as a defense mechanism against the oxidant, such as superoxide dismutase (SOD), catalase (CAT), and seleno-dependent glutathione peroxidase (GSH-Px) [20]. However, those antioxidant levels decrease when there is excessive free radical production [21].

### 4.1. SOD and MDA

The SOD activities were lower in rats exposed to TCDD (T0) than those of normal rats. TCDD activated the aryl hydrocarbon receptor (AhR) and led to dimerization with the aryl hydrocarbon receptor nuclear translocator (ARNT) and transcriptional activation of several xenobiotic-metabolizing enzymes, such as SOD. SOD was essential for protecting cells against the harmful superoxide radical derived from mitochondrial respiratory chain leakage. SOD causes the dismutation of the superoxide radicals to molecular oxygen and hydrogen peroxide [7, 13]. SOD inactivation was resulted from oxidative stress due to excessive reactive oxygen species (ROS) [22]. Administration of *α*-tocopherol, starting from 140 mg per kg BW, was sufficient to restore the SOD activity level to that of the normal rats. This may be due to the antioxidant of *α*-tocopherol scavenges excess free radicals in cells, preventing SOD inactivation [20].

The MDA level of rats exposed to TCDD were higher than those of normal rats. Polyunsaturated fatty acids of the cell membrane easily accepted unpaired electrons from ROS, causing lipid peroxidation [6]. The end product of lipid peroxidation was MDA; therefore, MDA was a biomarker for oxidative stress [23]. The increased MDA levels in rats exposed to TCDD reflect the level of damage to the plasma membrane attacked by free radicals. When ROS levels were higher than endogenous antioxidants (among others is SOD), membrane integrity was lost, and MDA levels increased. Administration of *α*-tocopherol starting from 77 mg per kg BW was sufficient to return MDA levels like those of normal rats. The result of this study was following the earlier report of the higher antioxidant level, followed by the lower MDA levels [24].

### 4.2. Live Spermatozoa

In the control group, about 14-15% of the spermatozoa population died. The normal metabolic activity could also cause death in the spermatozoa itself because the lactic acid produced lowered semen pH [25]. The rat sperm viability decreased along with increasing the dead, apoptotic, and necrotic spermatozoa due to the TCDD exposure. A substantial decrease in the quality of spermatozoa occurred due to a large number of dead spermatozoa [26]. Short-term exposure to TCDD has been shown to affect live spermatozoa [27]. TCDD elicits its effects by inducing AhR degradation and requires ubiquitination of the protein as a mechanism of mediated gene induction [28]. Sperm DNA fragmentation due to increased ROS affected live spermatozoa [29]. TCDD also inhibited the action of the 17-*β*-hydroxysteroid oxidoreductase enzyme in testosterone synthesis, causing a decrease in testosterone levels. Testosterone was essential in maintaining the viability of spermatozoa as long as they are stored in the epididymis [30–32].

*α*-tocopherol could protect the health of the mitochondrial plasma membrane to maintain the viability of spermatozoa [33]. In this study, a dose of 259 mg/kg BW/d *α*-tocopherol completely covered the effects of TCDD on sperm viability. The antioxidant effects of *α*-tocopherol worked not only at the spermatogenesis stage but also during endocrine regulation that maintains spermatozoa's viability [33]. *α*-tocopherol is a fat-soluble antioxidant, so it is more effective as a protector against oxidative stress [34], and it scavenges ROS and stops the chain propagation reaction in the lipid peroxidation process [9, 10] due to TCDD is also fat-soluble. Therefore, *α*-tocopherol is essential in maintaining the physiological integrity of the testicular cells, epididymis, and accessory glands that play essential roles in spermatogenesis and the maturation and viability of spermatozoa [35, 36]. These results were consistent with the findings of Ghafarizadeh et al. [37] that *α*-tocopherol increases sperm viability in vitro.

### 4.3. Necrosis and Apoptosis

Cell death could be caused by apoptosis and necrosis; both could be induced by toxic substances [38], including overproduction of ROS [39]. Staining with ethidium bromide and acridine orange in necrotic cells made all the cells appear brown [16, 40]. In apoptotic cells, not all dyes could enter the cell because the cell membrane was still intact, but the DNA was fragmented [41, 42] and stained so that the nucleus is yellow with brownish-red cytoplasm [16, 40]. Necrosis was an acute and irreversible cell death due to damage to the plasma membrane that could not maintain homeostasis to allow the entry of water and extracellular ions [43]. Cell necrosis may occur due to the activity of the lysozyme that digests the cell membrane's organelles, such as the mitochondrial membrane, ribosomes, and other cell apparatus, including intracytoplasmic fluid, followed by cell lysis [44]. However, the cell damage was reversible if the cause was immediately removed [45]. Apoptosis was a programmed cell death that was not preceded by cell swelling or inflammation. Apoptotic cells would shrink due to a breakup of the cell nucleus and chromosomes, which all form apoptotic bodies [46]. The mitochondrial membrane was rich in lipids sensitive to free radical attack [47]. In the mitochondrial membrane, there were Bcl-2 or Bcl-XL proteins that bound to Bax proteins that regulate the mechanism of apoptosis [48]. The apoptosis is triggered by lipid peroxidation, which leads to ROS activation in a continuous redox cycle. This cascade also includes the increased activity of a p53 protein, which will activate the Bax protein and then stimulate mitochondria to produce excessive cytochrome c that will cause apoptosis [44]. Cytochrome c is made up of apoptogenic proteins, such as the apoptosis-inducing factor and the endonuclease G [35], followed by caspase activation and exposure to phosphatidylserine on the surface of spermatozoa a few hours later [49]. The increasing ROS affected the damage of DNA, especially the integrity of DNA as a cause of cell death [50], and caused the limited cytoplasmic capacity for DNA repair. The higher DNA fragmentation correlated with the activation of caspase-3 [51] as the executioner of apoptosis [52]. Antioxidant effect of *α*-tocopherol at 259 mg/kg BW recovers live, dead, necrosis, and apoptosis of spermatozoa which returns to like those of normal rats.

An increase in MDA levels and a decrease in SOD indicated oxidative stress followed by cell death mechanisms, such as apoptosis or necrosis [53]. Administration of *α*-tocopherol started from a dose of 77 and 140 mg per kg BW, returning MDA levels and SOD activity to those of normal rats. However, a dose of 259 mg/kg BW *α*-tocopherol was needed to recover the percentage of living, dead, apoptosis, and necrosis of spermatozoa. This may be due to the effect of TCDD on sperm viability, and its recovery by *α*-tocopherol requires a more complicated mechanism and extended pathway than are needed to recover MDA levels and SOD activity. The maintaining of testicle health involves the dynamics of complex upregulation of the endocrine. The explanation of *α*-tocopherol role for recovery testicular damage due to TCDD exposure in this study was limited based on SOD and MDA levels. Therefore, further studies were needed to determine changes in GnRH, FSH, LH, and testosterone levels, including the role of *α*-tocopherol to prevent apoptosis of testicular tissue at gene level such as AhR, Bax, and Bcl casp3.

Necrosis is acute and irreversible cell death due to damage to the plasma membrane that cannot maintain homeostasis to allow the entry of water and extracellular ions [43]. Cell necrosis may occur due to the activity of the lysozyme that digests the cell membrane's organelles, such as the mitochondrial membrane, ribosomes, and other cell apparatus, including intracytoplasmic fluid; damage is caused to the cells, followed by cell lysis [44]; however, the cell damage is reversible if the cause is immediately removed [45]. Administration of *α*-tocopherol starts from a dose of 77 and 140 mg per kg BW, returning MDA levels and SOD activity to those of normal rats. MDA levels and SOD activity of rats exposed to TCDD and treated with *α*-tocopherol at dose 140 or 259 mg/kg BW daily were not statistically significant (*p* > 0.05) than those of normal rats (control group).

## 5. Conclusion

*α*-tocopherol rebalances SOD and MDA levels to recover spermatozoa's viability from necrosis and apoptosis death of male rats exposed to TCDD.

## Figures and Tables

**Table 1:** SOD activity (%) and MDA level (nm/mL) of rats exposed to TCDD and treated with *α*-tocopherol.

Group	SOD	MDA
C	73.66 ± 10.35^a^	4.17 ± 0.85^b^
T0	32.64 ± 3.12^c^	9.68 ± 1.65^a^
T1	56.32 ± 3.07^b^	3.24 ± 0.34^b^
T2	65.46 ± 2.73^a^	4.04 ± 0.61^b^
T3	68.27 ± 5.07^a^	4.61 ± 0.25^b^

**Table 2:** The percentage of live, dead, apoptotic, and necrotic spermatozoa (%) of rats exposed to TCDD and treated with *α*-tocopherol.

Group	Live spermatozoa	Dead spermatozoa	Apoptotic spermatozoa	Necrotic spermatozoa
C	85, 44 ± 3, 71^a^	14.56 ± 3, 97^c^	8, 32 ± 2, 26^c^	6, 24 ± 2, 00^c^
T0	33, 84 ± 4, 41^c^	66.16 ± 5, 05^a^	35, 43 ± 6, 05^a^	30, 73 ± 4, 31^a^
T1	65, 55 ± 4, 19^b^	34.45 ± 6, 62^b^	18, 44 ± 2, 41^b^	16, 01 ± 3, 54^b^
T2	72, 76 ± 4, 8a^b^	27.24 ± 6, 09^b^	14, 08 ± 3, 42^b^	13, 16 ± 2, 76^b^
T3	81, 82 ± 2, 93^a^	18.18 ± 3, 82^c^	9, 51 ± 2, 17^c^	8, 67 ± 1, 94^c^

## Data Availability

The data used to support the findings of this study are available from the first author.
